# Epidemiological characteristics and prevalence rates of research reproducibility across disciplines: A scoping review of articles published in 2018-2019

**DOI:** 10.7554/eLife.78518

**Published:** 2023-06-21

**Authors:** Kelly D Cobey, Christophe A Fehlmann, Marina Christ Franco, Ana Patricia Ayala, Lindsey Sikora, Danielle B Rice, Chenchen Xu, John PA Ioannidis, Manoj M Lalu, Alixe Ménard, Andrew Neitzel, Bea Nguyen, Nino Tsertsvadze, David Moher

**Affiliations:** 1 Heart Institute, University of Ottawa Ottawa Canada; 2 School of Epidemiology and Public Health, University of Ottawa Ottawa Canada; 3 Department of Anaesthesiology, Clinical Pharmacology, Intensive Care and Emergency Medicine, Geneva University Hospitals Geneva Switzerland; 4 Centre for Journalology, Clinical Epidemiology Program, Ottawa Hospital Research Institute Ottawa Canada; 5 School of Dentistry, Federal University of Pelotas Pelotas Brazil; 6 Gerstein Science Information Centre, University of Toronto Toronto Canada; 7 Health Sciences Library, University of Ottawa Ottawa Canada; 8 Department of Psychology, McGill University Montreal Canada; 9 Department of Medicine, University of Ottawa Ottawa Canada; 10 Departments of Medicine, of Epidemiology and Population Health, of Biomedical Data Science, and of Statistics, and Meta-Research Innovation Center at Stanford, Stanford University Stanford United States; 11 Department of Anesthesiology and Pain Medicine, University of Ottawa Ottawa Canada; 12 Regenerative Medicine Program, Ottawa Hospital Ottawa Canada; Indiana University United States; Icahn School of Medicine at Mount Sinai United States

**Keywords:** reproducibility, replication, meta-research, open science, None

## Abstract

**Background:**

Reproducibility is a central tenant of research. We aimed to synthesize the literature on reproducibility and describe its epidemiological characteristics, including how reproducibility is defined and assessed. We also aimed to determine and compare estimates for reproducibility across different fields.

**Methods:**

We conducted a scoping review to identify English language replication studies published between 2018 and 2019 in economics, education, psychology, health sciences, and biomedicine. We searched Medline, Embase, PsycINFO, Cumulative Index of Nursing and Allied Health Literature – CINAHL, Education Source via EBSCOHost, ERIC, EconPapers, International Bibliography of the Social Sciences (IBSS), and EconLit. Documents retrieved were screened in duplicate against our inclusion criteria. We extracted year of publication, number of authors, country of affiliation of the corresponding author, and whether the study was funded. For the individual replication studies, we recorded whether a registered protocol for the replication study was used, whether there was contact between the reproducing team and the original authors, what study design was used, and what the primary outcome was. Finally, we recorded how reproducibilty was defined by the authors, and whether the assessed study(ies) successfully reproduced based on this definition. Extraction was done by a single reviewer and quality controlled by a second reviewer.

**Results:**

Our search identified 11,224 unique documents, of which 47 were included in this review. Most studies were related to either psychology (48.6%) or health sciences (23.7%). Among these 47 documents, 36 described a single reproducibility study while the remaining 11 reported at least two reproducibility studies in the same paper. Less than the half of the studies referred to a registered protocol. There was variability in the definitions of reproduciblity success. In total, across the 47 documents 177 studies were reported. Based on the definition used by the author of each study, 95 of 177 (53.7%) studies reproduced.

**Conclusions:**

This study gives an overview of research across five disciplines that explicitly set out to reproduce previous research. Such reproducibility studies are extremely scarce, the definition of a successfully reproduced study is ambiguous, and the reproducibility rate is overall modest.

**Funding:**

No external funding was received for this work

## Introduction

Reproducibility is a central tenant of research. Reproducing previously published studies helps us to discern discoveries from false leads. The lexicon around reproducibility studies is diverse and poorly defined (Goodman et al., 2016). Here, we loosely use Nosek and Errington’s definition: ‘*a study for which any outcome would be considered diagnostic evidence about a claim from prior research’* (Nosek and Errington, 2020a). Most scientific studies are never formally reproduced and some disciplines have lower rates of reproducibility attempts than others. For example, in education research, an analysis published in 2014 of all publications in the discipline’s top 100 journals found that only 0.13% (221 out of 164,589) of the published articles described an independent reproducibility study (Makel and Plucker, 2014). There is rising concern about the reproducibility of research and increasing interest in enhancing research transparency (e.g. Buck, 2015; Munafò et al., 2017; Collins and Tabak, 2014; Begley and Ioannidis, 2015).

Knowledge about rates of reproducibility is currently dominated by a handful of well-known projects examining the reproducibility of a group of studies within a field. For example, a project estimating the reproducibility of 100 psychology studies published in three leading journals found that just 36% had statistically significant results, compared to 97% of the original studies. Just 47% of the studies had effect sizes that were within the bounds of the 95% confidence interval of the original study (Open Science Collaboration, 2015). Estimates of reproducibility in economics are similarly modest or low: a large-scale study attempting to reproduce 67 papers was only able to reproduce 22 (33%) of these (Chang and Li, 2015). This same study showed that when teams attempting to reproduce research involved one of the original study authors as a co-author on the project, rates of reproducibility increased. This may suggest that detailed familiarity with the original study method increases the likelihood of reproducing the research findings. A cancer biology reproducibility project launched in 2013 to independently reproduce several high-profile papers has produced rather sobering results a decade later. Most of the selected studies could not even be attempted to be reproduced (e.g. it was not clear what had been done originally) and among those that an attempt to reproduce was made, most did not seem to produce consistent results (although the exact reproducibility rate depends on the definition of reproducibility) and the effect of the reproduced studies was only 15% of the original effect (Errington et al., 2021a; Kane and Kimmelman, 2021; Errington et al., 2021b).

In medicine, studies that do not reproduce in clinic may exaggerate patient benefits and harms (Le Noury et al., 2015) especially when clinical decisions are based on a single study. Despite this and other potential consequences we know very little about what predicts research reproducibility. No data exists which provides systematic estimates for reproducibility across multiple disciplines or addresses why disciplines might vary in their rates of reproducibility. Failure to empirically examine reproducibility is regrettable: without research we can’t identify actions to take that could drive improvements in research reproducibility. This contributes to research waste (Nasser et al., 2017; Ioannidis et al., 2014; Freedman et al., 2015).

We set out to broadly examine the reproducibility literature across five disciplines and report on characteristics including how reproducibility is defined, assessed, and document prevalence rates for reproducibility. We focused on studies that explicitly described themselves as reproducibility or replication studies addressing reproductions of previously published work.

## Methods

Our study was conducted using the framework proposed by Arksey and O’Malley, 2005 and the related update by Levac et al., 2010, and follows a five stage process: (1) identifying the research question, (2) identifying relevant studies, (3) study selection, (4) charting the data, (5) collating, summarizing and reporting the results.

### Protocol registration and Open Science statement

This protocol was shared on the Open Science Framework prior to initiating the study (https://osf.io/59nw4/). We used the PRISMA-ScR (Tricco et al., 2018) checklist to guide our reporting. Study data and materials are also available on the Open Science Framework (https://osf.io/wn7gm/).

### Eligibility criteria

#### Inclusion criteria

We included all quantitative reproducibility studies within the fields of economics, education, psychology, health sciences and biomedicine that were published in 2018 or 2019. Definitions we established for each discipline can be found in Appendix 1. We included all studies that explicitly self-described as a replication or a reproducibility study in which a previously published quantitative study is referred to and conducted again. As per Nosek and Errington’s definition (Nosek and Errington, 2020a), we did not require that methods be perfectly matched between the original study and the replication if the author described the study as a replication. We excluded studies where the main intention of the work was not framed as a reproducibility project.

#### Exclusion criteria

We excluded complementary and alternative forms of medicine as defined by the National Institutes of Health’s National Center for Complementary and Integrative Health (https://www.nccih.nih.gov/) for feasibility based on pilot searches. We excluded literature that was not published in English for feasibility, or that described exclusively qualitative research. We excluded conference proceedings, commentaries, narrative reviews, systematic reviews (not original research), and clinical case studies. We also excluded studies that described a replication of a study but where the original study was reporting in the same publication.

### Information sources and search strategy

Our search strategy was developed by trained information specialists (APA, LS), and peer reviewed using the PRESS guideline (McGowan et al., 2016). We restricted our search to the years 2018 and 2019 in order to maintain feasibility of this study given our available resources for screening and data extraction. We searched the following databases: Medline via Ovid (1946–2020), Embase via Ovid (1947–2020), PsycINFO via Ovid (1806–2020), Cumulative Index of Nursing and Allied Health Literature – CINAHL (1937–2020), Education Source via EBSCOHost (1929–2020), ERIC via Ovid (1966–2020), EconPapers (inception – 2020), International Bibliography of the Social Sciences (IBSS) via ProQuest (1951–2020), and EconLit via EBSCOHost (1969–2020). We performed forward and backward citation analysis of articles included for data extraction in Scopus and Web of Science (platforms including Science Citation Index Expanded (SCI-EXPANDED) -–1900-present and Social Sciences Citation Index (SSCI) -–1900-present) to identify additional potential documents for inclusion. All searches are reported using PRISMA-S (Rethlefsen et al., 2021). For full search details please see Appendix 2. A related supplementary search was developed a priori in which we searched preprint servers and conducted forward and backward citation searching (Appendix 3).

### Selection of sources of evidence and data charting process

Search results from the databases were imported into Distiller SR, 2023 (Evidence Partners, Ottawa, Canada) and de-duplicated. Search results from the supplementary searching were uploaded into Endnote, de-duplicated, and then uploaded into DistillerSR for screening. Team members involved in study screening (KDC, CAF, MCF, DBR, CX, AN, BN, LS, NT, APA) initially screened the titles and abstracts of 50 records and then reviewed conflicts to ensure high level of agreement among screeners (>90%). After piloting was complete, all potentially relevant documents were screened in duplicate using the liberal accelerated method in which records move to full-text screening if one or more reviewers indicate unclear or yes with regards to potential inclusion and two reviewers were required to exclude a record. Then, all included documents were screened in duplicate to ensure they met all eligibility requirements. All conflicts were resolved by consensus or, when necessary, third-party arbitration (MML, DM). The study screening form can be found in Appendix 4 and 5.

### Data extraction

Two team members (MCF, CAF) extracted document characteristics. Prior to extraction a series of iterative pilot tests were done on included documents to ensure consistency between extractors. We extracted information including publication year, funding information (if funded, funder type), number of authors, ethics approval, study design, and open science practices (study registration, data sharing) from each included document. We also categorized each included documents based on its discipline area (e.g. Economics, Education, Psychology, Health Sciences, Biomedicine, or any combination of these fields) and whether a single original study was being reproduced or if the paper reported the results from reproducing more than one original study. When a single study was reproduced more than once (e.g. different labs all replicated one study) we classified this as a ‘single’ reproducibility study. We extracted what the stated primary outcome was. If there was no primary outcome stated, we recorded this, and extracted the first stated outcome described in each document. Finally, we extracted what the results of the reproducibility project as reported by the authors (replicated, not replicated, mixed finding) and categorized method by which the authors of each relevant document assess reproducibility (e.g. comparison of effect sizes, statistical significance from p-values). Where relevant we extracted p-values and related statistical information. This allowed us to test the proportion of reproducible results that were statistically significant. The study extraction form can be found in Appendix 6. In instances where documents described multiple sub-studies, we recorded this and then extracted information from all unique quantitative studies describing a reproducibility study.

### Piloting

Team members extracting data (CAF, MCF) performed a calibration pilot test prior to the onset of full-text screening and extraction. Specifically, a series of included documents were then extracted independently. The team then met to discuss differences in extraction between team members and challenges encountered before extracting from subsequent documents. This was repeated until consensus was reached. Extraction was then done by a single reviewer with a second reviewer doing quality control for all documents. Conflicts were resolved by consensus or, when necessary, third-party arbitration (KDC, DBR, MML, DM).

### Synthesis of results

SPSS 27 (Microsoft) (IBM Support, 2023) was used for data analysis. The characteristics of all included documents are presented using frequencies and percentages and described narratively. We report descriptive statistics where relevant. We then report frequency characteristics of the reproducibility studies, and which reproduced based on authors description of their findings (i.e. using the varied definitions of replication that exist in the literature), per discipline. Next, we describe how factors such as team size, team composition, and discipline relate to the reproducibility study. We compared these factors based on how authors defined reproducibility as well as based on the definition that results were statistically significant (at a conventional threshold of p<0.05).

Text-based responses (e.g. primary outcome) underwent content analysis and are described in thematic groups using frequencies and percentages. All content analysis was done by two independent investigators (CF, KDC) using Microsoft Excel.

## Results

### Open science

Data and materials are available on the Open Science Framework (https://osf.io/wn7gm/).

### Protocol amendments

In our protocol, we specified we would include all documents that explicitly self-describe themselves as a reproducibility or replication study. Over the course of the scoping review we encountered studies that described themselves as being a replication or reproducibility study, but in fact did not describe work that met this definition (e.g. a longitudinal study reporting a new cross sectional report of the data; a study with the goal of replicating a concept rather than a specific study). In these instances, we excluded the study despite the authors description that it was a reproducibility study. Studies describing themselves as being a replication but that explicitly specified that they used a novel method were also excluded, as they did not set out with the explicit goal of replicating previous research approach. We encountered a study that was included where the results were arranged by outcomes but not studies being replicated, in this instance we were unable to determine how the results corresponded to the studies the author listed they reproduced. To accommodate this, we modified our extraction form to include an item indicating that extraction of sub-study information was unclear and could not be performed. We had indicated we would record whether the research involved the study of humans or animals, and if so, how many. We did not include these items on the extraction form after piloting as we found reporting of N to be incomplete making accuracy challenging. We also do not present a re-analysis of the reproducibility studies where we recalculate the rate at which studies reproduce comparatively by discipline given the relatively small representation of disciplines outside of psychology and health science which accounted for 128 (72.3%) of the total studies.

### Selection of sources of evidence

**Figure 1.**
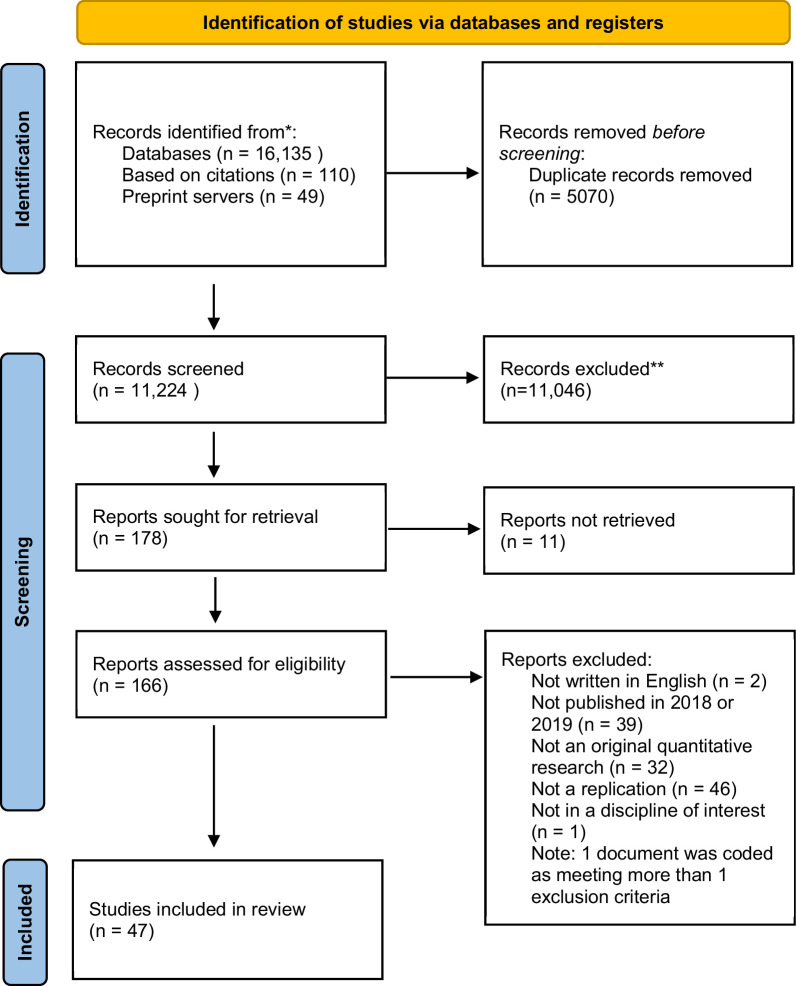
Flow diagram of articles.

The original search included 16,135 records. An additional 159 novel records were retrieved via grey literature searching: 7 documents were retrieved from searching citations of included documents, 49 were included from searching preprints servers, and 103 were included from citation searching. After de-duplication we screened a total of 11,224 documents, of which, 178 were sought for full-text screening. After full-text screening, 47 documents were included in the review. The remaining 131 documents were excluded because of one or more of these factors: they were not written in English (N=2), we could not obtain a full-text document via our library (N=11), the document was not published in 2018 or 2019 (N=39), the document did not describe an original quantitative research study (N=32), the study was not a quantitative reproduciblity study (N=46), or did not fit in a discipline of interest (N=1). See Figure 1 for the study flow diagram.

### Characteristics of sources of evidence

The characteristics of the included documents are summarized in Table 1. The 47 documents included described a total of 177 reproducibility studies. Thirty-six documents (76.6%) described a single reproducibility study, while 11 (23.4%) documents described multiple reproducibility efforts of distinct studies in a single paper. Twenty-eight (59.6%) documents were published in 2018 while 19 (40.4%) were published in 2019. The corresponding author on most of the documents was based at an institution in the USA 27 (57.4%). The included documents had a median of 3 authors, but papers ranged from having between 1 and 172 authors.

**Table 1.** Characteristics of included documents.

Characteristic	Categories	All studies	Single replication papers(N=36)	Multiple replication papers(N=11)
		N (%) (unless otherwise indicated)		
What discipline does the work best fit in?*	Biomedicine Economics Education Health sciences Psychology Other (mixture of two or more of the abov1e)	6 (3.4) 5 (2.8) 5 (2.8) 42 (23.7) 86 (48.6) 33 (18.6)	6 (16.7) 5 (13.9) 1 (2.8) 9 (25.0) 15 (41.7) -	- - 1 (9.1) 4 (36.4) 4 (36.4) 2 (18.2)
Year of publication	2018 2019	28 (59.6) 19 (40.4)	21 (58.3) 15 (41.7)	7 (63.6) 4 (36.4)
Country of corresponding author (reported based on Top 3 overall)	USA The Netherlands Australia	27 (57.4) 4 (8.5) 3 (6.4)	19 (52.8) 3 (8.3) 3 (8.3)	8 (72.7) 1 (9.1) -
Number of authors^†^	Median Range	3 1–172	3 1–124	4 1–172
Funding	Yes No Not reported	32 (68.1) 6 (12.8) 9 (19.1)	23 (63.9) 5 (13.9) 8 (22.2)	9 (81.8) 1 (9.1) 1 (9.1)
Funding source^‡^	Government Academic Non-profit Unsure	19 (59.4) 15 (46.9) 14 (43.8) 1 (3.1)	17 (73.9) 9 (39.1) 9 (39.1) -	2 (22.2) 6 (66.7) 5 (55.6) 1 (11.1)
Ethics approval	Yes No Ethics approval not relevant	23 (48.9) 10 (21.3) 14 (29.8)	17 (47.2) 8 (22.2) 11 (30.6)	6 (54.5) 2 (18.2) 3 (27.3)

* Data reported at the study level.

† Data reports median and range.

‡ Data refers to funded studies only, some studies report multiple funding sources.

Thirty-two (68.1%) documents indicated that they received funding, 6 (12.8%) indicated the work was unfunded, and 9 (19.1%) failed to report information about funding. Among documents reporting funding, federal governments were the primary source (N=19, 59.4%). Twenty-three (48.9%) studies reported receiving ethical approval, 10 (21.3%) studies did not report ethical approval, and ethical approval was not relevant for 14 (29.8%) studies.

### Synthesis of reproducibility studies

Most replication studies captured in our sample were in the discipline of psychology (86, 48.6%), followed by health science (42, 23.7%), or were an intersection of our included disciplines (33, 18.6%). There were a relatively smaller number of studies in economics (5, 2.8%), education (5, 2.8%), and biomedicine (6, 3.4%). The most common study designs observed were observational studies (85, 48.0%) and experimental studies (52, 29.4%). The remainder of studies captured were data studies for example, re-analysis using previous data (35, 19.8%) or experimental trials (5, 2.8%).

We examined whether the authors of the reproducibility studies included in our synthesis overlapped with the research team of the original studies. To do so, we compared of author lists and examined whether the authors of the reproducibility team self-report their team overlapped or had contact with the original author(s). Sixteen (9.0%) documents had teams that overlapped with the original research team whose study was being replicated, 44 (24.9%) indicated contact with the original team but not authorship overlap, while the remaining 117 (66.1%) studies had no authorship overlap and did not report any contact with the original study authors. Other key findings include that 81 (45.8%) of the studies referred to a registered protocol, although only 41 (23.2%) indicated they used a protocol that was identical to the original study they were reproducing and 28 (23.9%) had both a registered protocol and claimed to be identical to the original study. For 112 (63.3%) of studies the authors indicated that data of the replication studies was publicly available; however, this rate was driven by a few included documents that reported multiple reproduced studies and consistently shared data. Thirty-four of 47 (72.3%) documents included indicated data was not shared. Most studies did not report a primary outcome (134, 75.7%). For studies that did not list a primary outcome, where possible, we extracted the first reported outcome. We thematically grouped the primary/first stated outcomes of the remaining documents into 12 themes, which are presented in Appendix 8. Three of these themes were related to biomedicine or health. We present the data describing the characteristics of the included documents by discipline of the document in Table 2.

**Table 2.** Study replication methods characteristics.

Characteristic	Categories	All discipline studies(N=177)	BiomedicineN (%)	EconomicsN (%)	EducationN (%)	Health sciences*N (%)	PsychologyN (%)	Other (mixture of two or more of the above)N (%)
Did the replication study team specify that they contacted the original study project team?	Yes, the author teams overlapped Yes, there was contact No, the teams did not overlap or contact	16 (9.0) 44 (24.9) 117 (66.1)	2 (33.3) - 4 (66.7)	- - 5 (100)	- - 5 (100)	4 (9.5) 14 (33.3) 24 (57.1)	10 (11.6) 9 (10.5) 67 (77.9)	- 21 (63.6) 12 (36.4)
Does the replication study refer to a protocol that was registered prior to data collection?	Yes No	81 (45.8) 96 (54.2)	2 (33.3) 4 (66.7)	- 5 (100)	1 (20.0) 4 (80.0)	18 (42.9) 24 (57.1)	39 (45.3) 47 (54.7)	21 (63.6) 12 (36.4)
Do the authors specify that they used an identical protocol?	Yes No ^†^ Not reported Unsure	41 (23.2) 70 (39.5) 64 (36.2) 2 (1.1)	2 (33.3) 1 (16.7) - 2 (33.3)	1 (20.0) 3 (60.0) 1 (20.0) -	- 3 (60.0) 2 (40.0) -	9 (21.4) 15 (35.7) 17 (40.5) -	8 (9.3) 34 (39.5) 44 (51.2) -	21 (63.6) 12 (36.64) - -
Does the study indicate that data is shared publicly?	Yes^‡^ No	112 (63.3) 65 (36.7)	2 (33.3) 4 (66.7)	- 5 (100)	1 (20.0) 4 (80.0)	18 (42.9) 24 (57.1)	70 (81.4) 16 (18.6)	21 (63.6) 12 (36.4)
What is the study design used?	Data re-analysis Experimental Observational Trial	35 (19.8) 52 (29.4) 85 (48.0) 5 (2.8)	- 2 (33.3) 3 (50.0) 1 (16.7)	3 (60.0) 1 (20.0) 1 (20.0) -	1 (20.0) - 3 (60.0) 1 (20.0)	26 (61.9) 10 (23.8) 3 (7.1) 3 (7.1)	5 (5.8) 39 (45.3) 42 (48.8) -	33 (100) -
Did the study specify a primary outcome?	Yes No	43 (24.3) 134 (75.7)	- 6 (100)	- 5 (100)	- 5 (100)	26 (61.9) 16 (38.1)	13 (15.1) 73 (84.9)	- 33 (100)

* One study provided results by outcome not by studies being replicated, in this instance we were unable to determine how the results corresponded to the studies the author listed they replicated so these data are missing.

† In these instances authors specified deviations between their protocol and that of the original research team.

‡ This was not verified. We simply recorded what the authors reported. It is possible that self-reported sharing and rates of actual sharing are not identical.

### Definition

Table 3 provides a summary of definitions for reproducibility. We found that studies related to psychology and health sciences tended to use a comparison of effect sizes to define reproducibility success. The number of included studies across other disciplines was low (<6).

**Table 3.** Reproducibility characteristics of studies replicated overall and across disciplines.

Characteristic	Categories	Overall	Biomedicine	Economics	Education	Health sciences	Psychology	Other
How did the authors assess reproducibility?	Effect sizes Meta analysis of original effect size Null hypothesis testing using p-value Subjective assessment Other	116 (65.5) 33 (18.6) 17 (9.6) 5 (2.8) 6 (3.4)	1 (16.7) 2 (33.3) 2 (33.3) - 1 (16.7)	1 (20.0) - 2 (40.0) 1 (20.0) 1 (20.0)	1 (20.0) - - 2 (40.0) 2 (40.0)	25 (59.5) 9 (21.4) 5 (11.9) 2 (4.8) 1 (2.4)	76 (88.4) 1 (1.2) 8 (9.3) - 1 (1.2)	12 (36.4) 21 (63.6) - - -
Based on the authors definition of reproducibility, did the study replicate?	Yes No Mixed Unclear	95 (53.7) 36 (20.3) 8 (4.5) 38 (21.5)	4 (66.7) 1 (16.7) 1 (16.7) -	4 (80.0) 1 (20.0) - -	2 (40.0) 2 (40.0) 1 (20.0) -	36 (85.7) 4 (9.5) 1 (2.4) 1 (2.4)	25 (29.1) 19 (22.1) 5 (5.8) 37 (43.0)	24 (72.7) 9 (27.3) - -
Was the p-value reported on the statistical test conducted on the primary outcome?	Yes No/unclear	116 (65.5) 61 (34.5)	3 (50.0) 3 (50.0)	3 (60.0) 2 (40.0)	4 (80.0) 1 (20.0)	33 (78.6) 9 (21.4)	45 (52.3) 41 (47.7)	28 (84.8) 5 (15.2)

### Prevalence of reproducibility

Of the 177 individual studies reproduced, based on the authors reported definition, 95 (53.7%) reproduced, 36 (20.3%) failed to reproduce, 8 (4.5%) produced mixed results. A further 38 studies (21.5%), 37 of which were from a single included document, could not be assessed due to issues with incomplete or poor-quality reporting. Rates were highest in health sciences (N=36, 85.7%), economics (N=4, 80%), inter-disciplinary studies (N=24, 72.7%). Rates of replication tended to be lower in biomedicine (N=4, 66.7%), education (N=2, 40%), and psychology (N=25, 29.1%). When we removed an included document related to psychology, which presented 37 individual studies but failed to report a reproducibility outcome clearly, rates improved to 51.0% in this discipline. When examining the 35 studies that reported data (re)-analysis projects, rates of reproducibility based on the authors definition were considerably higher (N=31, 88.6%).

Of the 177 individual studies, we were able to extract p-values from 116 (65.5%), of these, reproducibility was statistically significant at the p<0.05 threshold in 82 (70.9%) studies.

## Discussion

The primary objective of this study was to describe the characteristics of reproducibility studies, including how reproducibility is defined and assessed. We found 47 individual documents reporting reproducibility studies in 2018–2019 that met our inclusion criteria. Our included documents reported 177 individual reproducibility studies. Most reproducibility studies were in the disciplines of psychology and health science (>72%), with 86 and 42 studies, respectively. This may suggest unique cultures around reproducibility in distinct disciplines, future research is needed to determine if such differences truly exist given the limitations of our search and approach. Some disciplines have routinely embedded replication as part of the original discovery and validation efforts, for example replications are routinely done for genomics and other -omics findings as part of the original studies rather than as separate efforts. Consistent with previous research (Makel et al., 2012; Sukhtankar, 2017; Hubbard and Vetter, 1992), our findings suggest that overall only a very small fraction of research in any of these discipline published in a given year focuses on reproducing existing research published in previous papers. A recent evaluation of 349 randomly selected research articles from the biomedical literature published in 2015-2018 (Serghiou et al., 2021) found that 33 (10%) included a reproducibility component in their research (e.g. validating previously published experiments, running a similar clinical trial in a different population, etc.). However, the vast majority of these efforts would not qualify as separate, independent replication studies in our assessment.

Most of the documents included in our study had corresponding/lead authors who were from the United States (57.4%) and most papers reported receiving funding (68.1%); papers reporting multiple studies (N=9, 81.8%) were more likely to report funding than single replication studies (N=23, 63.9%). Together this may suggest that at least some funders recognize the value of reproducibility studies, and that USA based researchers have taken a leadership role in reproducibility research. We note that just 11 of the 47 papers reported to be reproducing more than one original study, suggesting most reproducibility studies reproduce a single previous study. We note that two of the ‘single studies’ included reported being part of a larger reproducibility project (e.g. a study part of the broader cancer reproducibility project). Four of ‘single studies’ reported more than 1 reproduction of the same original article in the document (e.g. different labs reproducing the same experiment). Future qualitative research could shed light on the motivations of researchers to conduct a single versus multiple reproducibility study. This will be important to understand what, if any, supports are needed to facilitate large-scale reproducibility studies.

When we examined the 177 individual studies reproduced in the 47 documents, we found only a minority of them referred to registered protocols. In psychology, rates were highest, with 45.8% of studies referring to a registered protocol. Registered protocols are a core open science practice, they can help to enhance transparency, mitigate publication bias and selective reporting biases (Nosek et al., 2018; Nosek et al., 2019). Importantly, they may specify what analyses were planned a priori and which were post hoc. Registration would seem especially relevant to reproducibility projects in order to pre-specify approaches to reduce perceptions of bias. We acknowledge, however, that mandates for registration are rare and exist only in particular disciplines and for specific study designs.

There was a wide range of study designs. For example, in psychology observational (e.g. cohort study) and experimental studies dominated, while data analysis studies (e.g. re-analysis) were most prominent in health sciences and economics. Reproducing an observational or experimental study may pose a greater resource challenge as compared to reproducing a data analysis, which may explain the higher rates of reproducibility success observed among data analysis studies. When no new data are generated, it may be difficult in the current research environment, which tends to favor novelty, to publish a re-analysis of existing data that shows the exact same result (Ebrahim et al., 2014; Naudet et al., 2018).

Across our five disciplines of interest the norm was that author teams did not overlap or contact the team of the original study they were attempting to reproduce. This finding may not be generalizable, because by definition we did not consider documents where the original study was reproduced within the same paper, a practice that is commonplace in many disciplines, for example genomics. Nosek and Errington describe confusion and disagreement that occurred during large scale reproducibility projects they were involved in which produced results that failed to replicate the original findings, calling for original researchers and those conducting reproducibility projects to “argue about what a replication means before you do it”. Nosek and Errington, 2020b Our finding that teams don’t overlap or communicate, suggests that this practice is not typically implemented, despite its potential value to improve reproducibility Chang and Li, 2015. Conversely, involvement of the original authors as authors in the reproducibility efforts may increase the impact of allegiance and confirmation biases. In the published experience from the Reproducibility Project: Cancer Biology, a large share of original authors did not respond to efforts to reach them to obtain information about their study (Errington et al., 2021a; Kane and Kimmelman, 2021; Errington et al., 2021b).

Of the 177 individual studies reproduced, based on the authors’ reported definition, 53.7% reproduced successfully. When examining definitions for reproducibility, we found that studies related to psychology and health sciences tended to use a comparison of effect sizes to define reproducibility success. The number of included studies across other disciplines was too low to yield meaningful comparison of differences in definitions across disciplines. Rates of reproducibility based on the authors definition were highest in health sciences (N=36/42, 85.7%; 24/33, 72.7%) including ‘other’ and lower in biomedicine (N=4/6, 66.7%), education (N=2/5, 40%), and psychology (N=25, 29.1%). Low rates in psychology were driven by a single document reporting 37 studies that failed to report outcomes. When this document was removed, rates improved to 51.0% in this discipline. When we applied p-values from the 116 studies where these were reported, 70.7% of studies had p-values less than the commonly used 0.05 threshold. There is an increasing literature of different definitions of reproducibility and ‘success’ rates will unavoidably depend on how replication is defined (Errington et al., 2021a; Errington et al., 2021b; Held et al., 2022; Pawel and Held, 2020).

To our knowledge, this is the first study to provide a broad comparison of the characteristics of explicit reproducibility studies across disciplines. This comparative approach may help to identify features to better support further reproducibility research projects. This study used a formal search strategy, including grey literature searching, to identify potential documents. It is possible that the terminology we used, which was broad to apply across disciplines, may not have captured all potential studies in this area. It is also possible that the databases used do not equally represent the distinct disciplines we investigated, meaning that the searches are not directly comparable cross-disciplinarily. We also were not able to locate the full-text of all included documents which may have impacted on the results. The impact of these missing texts may not have been equal across disciplines. For these reasons, generalizations about disciplines should not be made. A further limitation is that we only considered two years of research. This allowed for a contemporary view on characteristics of replication studies, but it prevented the ability to examine temporal changes. Indeed, several well-known large-scale replication studies would not have been captured in our search. Ultimately, the number of included documents in some disciplines was relatively modest, suggesting that inclusion of articles across a larger timeframe is needed to address the objective to compare more meaningfully across disciplines.

For feasibility we also only extracted information about the primary outcome listed for each paper, or if no primary outcome was specified, the first listed outcome. It is possible that rates of reproducibility differ across outcomes. Future research could consider all outcomes listed. While we conducted our screening and extraction using two reviewers, to foster quality control, the reporting of the studies captured was sometimes extremely poor. This impacted the extraction process as in some cases extraction was challenging and in others resulted in missing data. Further, our screeners and extractors were not naïve to the aims of the study, which may have created implicit bias. Future research could include training coders and extractors who are unaware of the project aims. Collectively these study design decisions and practical challenges present limitations on the overall generalizability of the findings beyond our dataset. Finally, we acknowledge that these explicit ‘reproducibility check’ documents that we targeted, are only one part of the much larger scientific literature where some reproducibility features may be embedded. Random samples of biomedical papers with empirical data published in 2015–2018 have shown that reproducibility concepts are not uncommon (Wallach et al., 2018). In the psychological sciences, similarly 5% of a random sample of 188 papers with empirical data published in 2014–2017 were replications (Hardwicke et al., 2020). Conversely, in the social sciences, among a random sample of 156 papers published in 2014–2017, only 2 were replication studies (1%) (Hardwicke et al., 2020). Moreover, as mentioned above, some fields explicitly require replications to be included as part of the original publication, and the large and blossoming literature of tens of thousands of meta-analyses (Ioannidis, 2016) suggests that for many topics there are multiple studies that address similar enough questions so that meta-analysts would combine them. Eventually, the relative advantages and disadvantages of different replication practices (e.g. reproducibility embedded in the original publication versus done explicitly in a subsequent stage versus done as part of a wider agenda that mixes replication and novel efforts) needs further empirical study in diverse scientific fields.

Our finding that, only about half of the reproducibility studies reproduced across five fields of interest is concerning, though consistent with other studies. These estimates may not necessarily represent appropriately the reproducibility rates of entire fields since the choice of what specific studies to try to replicate may include selection factors that introduce strong bias towards higher or lower replication rates. Moreover, while estimates of reproducibility vary across fields in our modest sample, so too do norms in definitions used to define reproducibility. Choice of these definitions (especially when these definitions are not clear, pre-specified and valid) mayaffect the interpretation of these results to fit various narratives of replication success or failure. This suggests the need for discipline and interdisciplinary specific exchange on how to best approach reproducibility studies. Discussion on definitions for reproducibility, but also about methodological best practices when conducting a reproducibility study (e.g. using registered reports) will help to foster integrity and quality. To ensure reliability, multiple and diverse reproducibility studies with converging evidence are needed. At present, and as illustrated by out sampling, explicit reproducibility studies done as targeted reproducibility checks are rare. To enhance research reliability, reproducibility studies need to be encouraged, incentivized, and supported.

## Data Availability

Data and materials are available on the Open Science Framework (https://osf.io/wn7gm/). The following dataset was generated: CobeyKD 2022Scoping review data on reproducibility of research in a 2018-2019 multi-discipline sampleOpen Science Framework10.17605/OSF.IO/WN7GM

## Additional files

MDAR checklist

## Appendix 1

### Operationalized definitions of included disciplines

**Table:**

Discipline	Definition
Health sciences	Nutritional sciences, physiotherapy, kinesiology, rehabilitation, speech language pathology, physiology, nursing, midwifery, occupational therapy, social work, medicine and all its specialties, public health, population health, global health, pathology, laboratory medicine, optometry, health services research,
Biomedicine	Neuroscience, pharmacology, radiation therapy, dentistry, health management, epidemiology, virology, biomedicine, clinical engineering, biomedical engineering, genetics,
Education	Higher education, adult education, K-12 education, medical education, health professions education
Psychology	All specializations in Psychology, including but not limited to: clinical psychology, group psychology, psychotherapy, counselling, industrial psychology, cognitive psychology, forensic psychology, health psychology, neuropsychology, occupational psychology, social psychology
Economics	Microeconomics, macroeconomics, behavioural economics, econometrics, international economics, economic development, agricultural economics, ecological economics, environmental economics, natural resource economics, economic geography, location economics, real estate economics, regional economics, rural economics, transportation economics, urban economics, capitalist systems, comparative economic systems, developmental state, economic systems, transitional economies, economic history, industrial organization

## Appendix 2

### Search Strategy

1. exp "reproducibility of results"/
2. ((reproduc* or replicat* or reliabilit* or repeat* or repetition) adj2 (result* or research* or test*)).tw,kf.
3. (face adj validit*).tw,kf.
4. (test adj reliabilit*).tw,kf.
5. or/1–4
6. prevalence/
7. (prevalen* or rate or rates or recur* or reoccuren*).tw,kf.
8. 6 or 7
9. 5 and 8
10. limit 9 to (yr="2018–2019" and english)

### PRISMA-S Checklist

**Table:**

Section/topic	#	Checklist item	Location(s) Reported
INFORMATION SOURCES AND METHODS			
Database name	1	Name each individual database searched, stating the platform for each.	~line 185–190
Multi-database searching	2	If databases were searched simultaneously on a single platform, state the name of the platform, listing all of the databases searched.	n/a
Study registries	3	List any study registries searched.	n/a
Online resources and browsing	4	Describe any online or print source purposefully searched or browsed (e.g., tables of contents, print conference proceedings, web sites), and how this was done.	~line 195
Citation searching	5	Indicate whether cited references or citing references were examined, and describe any methods used for locating cited/citing references (e.g., browsing reference lists, using a citation index, setting up email alerts for references citing included studies).	~line 190–194
Contacts	6	Indicate whether additional studies or data were sought by contacting authors, experts, manufacturers, or others.	See appendix 3
Other methods	7	Describe any additional information sources or search methods used.	n/a
SEARCH STRATEGIES			
Full search strategies	8	Include the search strategies for each database and information source, copied and pasted exactly as run.	See appendix 2
Limits and restrictions	9	Specify that no limits were used, or describe any limits or restrictions applied to a search (e.g., date or time period, language, study design) and provide justification for their use.	
Search filters	10	Indicate whether published search filters were used (as originally designed or modified), and if so, cite the filter(s) used.	n/a
Prior work	11	Indicate when search strategies from other literature reviews were adapted or reused for a substantive part or all of the search, citing the previous review(s).	n/a
Updates	12	Report the methods used to update the search(es) (e.g., rerunning searches, email alerts).	n/a
Dates of searches	13	For each search strategy, provide the date when the last search occurred.	~line 190–194
PEER REVIEW			
Peer review	14	Describe any search peer review process.	~line 182
MANAGING RECORDS			
Total Records	15	Document the total number of records identified from each database and other information sources.	Figure 1
Deduplication	16	Describe the processes and any software used to deduplicate records from multiple database searches and other information sources.	~line 200–203
PRISMA-S: An Extension to the PRISMA Statement for Reporting Literature Searches in Systematic Reviews. Rethlefsen ML, Kirtley S, Waffenschmidt S, Ayala AP, Moher D, Page MJ, Koffel JB, PRISMA-S Group. Last updated February 27, 2020.			

## Appendix 3

### Grey literature search approach

1. Search reference lists of articles included for data extraction.
2. Forward/backward citation analysis of articles included for data extraction in Scopus and Web of Science (platforms including Science Citation Index Expanded (SCI-EXPANDED) -–1900-present and Social Sciences Citation Index (SSCI) -–1900-present).
3. Google Scholar search as follows: “Reproducibility” limited to the years 2018–2019.

Search of the following preprint servers: OpenScience Framework (OSF) including OSF Preprints, bioRxiv, EdArXiv, MediArXiv, NutriXiv, PeerJ, Preprints.org, PsyArXiv and SocArXiv, NBER Working Papers, Munich Personal RePEc Archive

## Appendix 4

### Level 1 Screening form item and criteria

**Table:**

1. ISSUE: Does the study self-report to be a replication of previous quantitative research? (yes/no/unsure)	
**INCLUDE**	**EXCLUDE**
All research articles containing one or more replications of quantitative research studies.	All research articles not describing a replication study All research articles describing exclusively qualitative replication studies

## Appendix 5

### Level 2 Screening form

**Table:**

1. LANGUAGE – Is this study in English? (yes/no/unsure)	
**INCLUDE**	**EXCLUDE**
Studies written in English.	Studies written in any other language that is not English.

**Table:**

2. DATE– Is this study published in 2018 or 2019? Use the most recent year stated on the publication (yes/no/unsure)	
**INCLUDE**	**EXCLUDE**
Studies published in 2018 or 2019.	Studies publisher in any other year. EPub ahead of print.

**Table:**

3. PUBLICATION TYPE – Is this the right publication type? (yes/no/unsure)	
**INCLUDE**	**EXCLUDE**
Original research articles describing quantitative research.	Narrative reviews Scoping reviews Systematic reviews Realist reviews Mapping reviews Literature reviews Rapid reviews Meta-Analysis Overview or reviews Umbrella reviews In short any type of literature review or synthesis should be excluded. Conference proceedings Book chapters Editorials, letters to the editor, commentaries Opinion pieces Case reports Case control studies Case series Protocols Guidelines Web pages Thesis projects Policy documents All exclusively qualitative research studies

**Table:**

4. ISSUE: Does the study self-report to be a replication of previous quantitative research? (yes/no/unsure)	
**INCLUDE**	**EXCLUDE**
All research articles containing one or more replications of quantitative research studies.	All research articles not describing a replication study All research articles describing exclusively qualitative replication studies

**Table:**

5. DISCIPLINE – Does the study focus education, economics, psychology, biomedicine or health sciences? (yes/no/unsure)	
**INCLUDE**	**EXCLUDE**
All research that is related to the disciplines of education, economics, psychology, biomedicine or health sciences. (Table 1 info to be provided).	All research that is not related to education, economics, psychology, biomedicine or health sciences Exclude research related to complementary and alternative medicine.

## Appendix 6

### Level 3 extraction form

1. Year of publication (use the most recent year stated on the document):
2. Name of the journal/outlet the document is published in (Do not use abbreviations):
3. Corresponding author e-mail (If there is more than one corresponding author indicated, extract the first listed author only. Extract the name in the format: Initial Surname, e.g., D Moher. If there is more than one e-mail listed, extract the first listed e-mail only.):
4. Country of corresponding author affiliation (If there is more than one corresponding author indicated, extract the first listed corresponding author only. If there is more than one affiliation listed for this individual, extract from the first affiliation only):
5. How many authors are named on the document?
6. Does the study report a funding source (yes, no)
  - If yes, which type of funder. Check all that apply. (Government, Academic, Industry, Non-Profit, Other, Can’t tell)
7. Did the study report ethics approval (yes, no, ethics approval not relevant)
8. Did the study recruit human participants or use animal participants? (yes, no)
  - If yes; Does the study involve humans or animals? (humans, animals)
  - If human; Please specify how many were involved? (use the total number of participants enrolled, not necessarily the number analyzed):
  - If animal; Please specify how many were involved? (use the total number of animals included, not necessarily the number analyzed):
9. Did the replication study team specify that they contacted the original study project team? (Yes, the author teams were reported to overlap; Yes, there was contact but author teams do not overlap; No, the replication team did not report any interaction)
10. Does the replication study refer to a protocol that was registered prior to data collection? (yes, no)
11. Does the study indicate that data is shared publicly? (yes, no)
12. How many quantitative replication studies are reported in the paper? (One study, more than one study).

Note: If more than one study is reported, we will extract information from each quantitative study using the following questions.

1. What is the study design used: (observational study; clinical trial; experimental; data analysis, other):
2. Did the study specify a primary outcome being? (Yes/No).
3. If a primary outcome was stated, what was it? If a primary outcome was not stated, please extract that first stated outcome described in the study results section (note these will be thematically grouped).
4. What discipline does this work best fit in? (Economics, Education, Psychology, Health Sciences, Biomedicine, Other)
5. How did the authors of the study assess reproducibility?
  - **Evaluating against the null hypothesis:** determining whether the replication showered a statistically significant effect, in the same direction as the original study, with a *P*-value <0.05.
  - **Effect sizes:** Evaluating replication effect against original effect size to examine for differences.
  - **Meta-analysis of original effect size:** Evaluates effect sizes considering variance and of 95% confidence intervals.
  - **Subjective assessment of replication:** An evaluation made by the research team as to whether they were successful in replicating the study findings.
  - **Other, please specify**.
  - **Unclear**
6. Based on the authors definition of reproducibility, did the study replicate? (yes, no, mixed)
7. What was the p-value reported on the statistical test conducted on the main outcome? (value:; not reported)
8. What was the effect size reported on the statistical test conducted on the main outcome? Size: Measure:

## Appendix 7

### List of included documents

**Table:**

ID	Year	Journal	Corresponding Author	Funding	Number of studies replicated	Discipline
20131	2018	Advances in Methods and Practices in Psychological Science	RJ McCarthy	Yes	One study	psychology
20130	2018	Advances in Methods and Practices in Psychological Science	B Verschuere	Yes	One study	psychology
20128	2018	Association for Psychological Science	M O'Donnell	Yes	One study	psychology
20126	2019	Association for Psychological Science	CJ Soto	Yes	More than one study	psychology
20125	2018	Psychological Science	TW Watts	Yes	One study	psychology
20124	2018	eLife	MS Nieuwland	Yes	One study	psychology
20122	2019	Journal of Environmental Psychology	S Van der Linden	Not reported	One study	psychology
20120	2019	The European Political Science Association	A Coppock	Yes	More than one study	other
20119	2018	Finance Research Letters	dirk.baur@uwa.edu.au	Not reported	One study	economics
20094	2019	Journal of Economic Psychology	AK Shah	Yes	More than one study	psychology
20048	2018	bioRxiv	X Zhang	Yes	One study	biomedicine
20001	2018	Royal society open science	T Schuwerk	Yes	More than one study	psychology
10805	2018	Rehabilitation Counseling Bulletin	BN Philips	Yes	One study	economics
20000	2018	Advances in Methods and Practices in Psychological Science	RA Klein	Yes	More than one study	psychology
13598	2018	Molecular Neurobiology	A Chan	Yes	One study	biomedicine
13165	2018	Journal of Contemporary Criminal Justice	JP Stamatel	No	One study	psychology
12923	2019	PLOS One	LM Smith	Yes	One study	health sciences
12287	2019	J Autism Dev Disord	L K Fung	Yes	One study	psychology
11535	2018	Journal of Obsessive-Compulsive and Related Disorders	EN Riise	No	One study	psychology
11456	2018	eLife	J Repass	Yes	One study	biomedicine
11003	2018	Journal of Health Economics	D Powell	Yes	One study	health sciences
10567	2019	Personality and Individual Differences	JJ McGinley	No	One study	health sciences
10555	2019	J Nerv Ment Dis	G Parker	Yes	One study	psychology
9886	2018	The BMJ	J P A Ioannidis	No	More than one study	health sciences
9505	2019	BMC Geriatrics	S E Straus	Yes	One study	health sciences
9193	2018	Journal for Research in Mathematics Education	K Melhuish	Not reported	More than one study	education
9011	2018	Journal of Contemporary Criminal Justice	CD Maxwell	Yes	One study	psychology
8574	2018	Public Opinion Quarterly	J A Krosnick	Yes	One study	economics
6213	2019	Psychophysiology	M Arns	No	One study	biomedicine
5825	2019	BMC Geriatrics	J.Holroyd-Leduc	Yes	One study	health sciences
5539	2018	Empirical Economics	B.Hayo	Not reported	One study	economics
5458	2018	Reproducing Public Health Services and Systems Research	J K Harris	Yes	More than one study	health sciences
5138	2019	Behavior Therapy	E J Wolf	Yes	One study	psychology
5133	2019	Brain, Behavior, and Immunity	FR Guerini	Yes	One study	biomedicine
5100	2019	European Neuropsychopharmacology	R Lanzenberger	Yes	One study	biomedicine
4794	2018	American Psychological Association	R J Giuliano	Not reported	One study	health sciences
3962	2019	Journal of Applied Behaviour Analysis	G Rooker	Not reported	One study	health sciences
3677	2019	Journal of Pediatric Psychology	B D Earp	Yes	One study	psychology
3574	2019	Personnel Psychology	G F Dreher	Not reported	One study	psychology
3138	2018	Journal of Second Language Writing	C de Kleine	Yes	One study	education
2310	2019	PLOS ONE	B Chen	Yes	More than one study	health sciences
1959	2018	Nature Human Behaviour	BA Nosek	Yes	More than one study	other
1837	2018	Oxford Bulletin Of Economics and Statistics	D Buncic	Not reported	One study	economics
1681	2018	Cortex	SG Brederoo	Yes	More than one study	health sciences
1477	2019	Frontiers in Psychology	M Boch	Yes	One study	psychology
727	2018	Archives of Clinical Neuropsychology	P Armistead-Jehle	No	One study	health sciences
584	2018	Australian Psychologist	RJ Brunton	Not reported	One study	health sciences

## Appendix 8

### Thematic groups of primary outcomes of studies replicated

**Table:**

No.	Theme	N (%)
1	Clinical and biological outcomes	19 (10.7)
2	Public health	5 (2.8)
3	Mental health and wellbeing	6 (3.4)
4	Criminology	5 (2.8)
5	Economics	8 (4.5)
6	Individual differences	58 (32.8)
7	Visual cognition	11 (6.2)
8	Morality	5 (2.8)
9	Score and performance	11 (6.2)
10	Political views	11 (6.2)
11	Not reported/unclear	30 (17.0)
12	Other	8 (4.5)

## References

[bib1] Arksey H, O’Malley L (2005). Scoping studies: towards a methodological framework. International Journal of Social Research Methodology.

[bib2] Begley CG, Ioannidis JPA (2015). Reproducibility in science: improving the standard for basic and Preclinical research. Circulation Research.

[bib3] Buck S (2015). Solving reproducibility. Science.

[bib4] Chang AC, Li P (2015). Is Economics Research Replicable? Sixty Published Papers from Thirteen Journals Say”Usually Not”.

[bib5] Collins FS, Tabak LA (2014). NIH plans to enhance reproducibility. Nature.

[bib6] Distiller SR (2023). DistillerSR: Literature Review Software Smarter Reviews: Trusted Evidence. https://www.evidencepartners.com/products/distillersr-systematic-review-software/.

[bib7] Ebrahim S, Sohani ZN, Montoya L, Agarwal A, Thorlund K, Mills EJ, Ioannidis JPA (2014). Reanalyses of randomized clinical trial data. JAMA.

[bib8] Errington TM, Denis A, Perfito N, Iorns E, Nosek BA (2021a). Challenges for assessing Replicability in Preclinical cancer biology. eLife.

[bib9] Errington TM, Mathur M, Soderberg CK, Denis A, Perfito N, Iorns E, Nosek BA (2021b). Investigating the Replicability of Preclinical cancer biology. eLife.

[bib10] Freedman LP, Cockburn IM, Simcoe TS (2015). The Economics of reproducibility in Preclinical research. PLOS Biology.

[bib11] Goodman SN, Fanelli D, Ioannidis JPA (2016). What does research reproducibility mean?. Science Translational Medicine.

[bib12] Hardwicke TE, Wallach JD, Kidwell MC, Bendixen T, Crüwell S, Ioannidis JPA (2020). An empirical assessment of transparency and reproducibility-related research practices in the social sciences (2014–2017). Royal Society Open Science.

[bib13] Held L, Matthews R, Ott M, Pawel S (2022). Reverse-Bayes methods for evidence assessment and research synthesis. Research Synthesis Methods.

[bib14] Hubbard R, Vetter DE (1992). The publication incidence of Replications and critical commentary in economics. The American Economist.

[bib15] IBM Support (2023). IBM SPSS Statistics 26. https://www.ibm.com/support/pages/downloading-ibm-spss-statistics-26.

[bib16] Ioannidis JPA, Greenland S, Hlatky MA, Khoury MJ, Macleod MR, Moher D, Schulz KF, Tibshirani R (2014). Increasing value and reducing waste in research design, conduct, and analysis. The Lancet.

[bib17] Ioannidis JPA (2016). The mass production of redundant, misleading, and Conflicted systematic reviews and meta-analyses. The Milbank Quarterly.

[bib18] Kane PB, Kimmelman J (2021). Is Preclinical research in cancer biology reproducible enough. eLife.

[bib19] Le Noury J, Nardo JM, Healy D, Jureidini J, Raven M, Tufanaru C, Abi-Jaoude E (2015). Restoring study 329: Eficacy and harms of paroxetine and Imipramine in treatment of major depression in adolescence. BMJ.

[bib20] Levac D, Colquhoun H, O’Brien KK (2010). Scoping studies: advancing the methodology. Implementation Science.

[bib21] Makel MC, Plucker JA, Hegarty B (2012). Replications in psychology research: how often do they really occur. Perspectives on Psychological Science.

[bib22] Makel MC, Plucker JA (2014). Facts are more important than novelty: replication in the education sciences. Educational Researcher.

[bib23] McGowan J, Sampson M, Salzwedel DM, Cogo E, Foerster V, Lefebvre C (2016). Guideline statement PRESS peer review of electronic search strategies: 2015 guideline statement. Journal of Clinical Epidemiology.

[bib24] Munafò MR, Nosek BA, Bishop DVM, Button KS, Chambers CD, du Sert NP, Simonsohn U, Wagenmakers EJ, Ware JJ, Ioannidis JPA (2017). A manifesto for reproducible science. Nature Human Behaviour.

[bib25] Nasser M, Clarke M, Chalmers I, Brurberg KG, Nykvist H, Lund H, Glasziou P (2017). What are Funders doing to minimise waste in research? lancet. Lancet.

[bib26] Naudet F, Sakarovitch C, Janiaud P, Cristea I, Fanelli D, Moher D, Ioannidis JPA (2018). Data sharing and Reanalysis of randomized controlled trials in leading BIOMEDICAL journals with a full data sharing policy: survey of studies published in *the BMJ* and *PLOS Medicine*. BMJ.

[bib27] Nosek BA, Ebersole CR, DeHaven AC, Mellor DT (2018). The Preregistration revolution. PNAS.

[bib28] Nosek BA, Beck ED, Campbell L, Flake JK, Hardwicke TE, Mellor DT, van ’t Veer AE, Vazire S (2019). Preregistration is hard, and worthwhile. Trends in Cognitive Sciences.

[bib29] Nosek BA, Errington TM (2020a). What is replication. PLOS Biology.

[bib30] Nosek BA, Errington TM (2020b). The best time to argue about what a replication means? before you do it. Nature.

[bib31] Open Science Collaboration (2015). PSYCHOLOGY. Estimating the reproducibility of psychological science. Science.

[bib32] Pawel S, Held L (2020). Probabilistic forecasting of replication studies. PLOS ONE.

[bib33] Rethlefsen ML, Kirtley S, Waffenschmidt S, Ayala AP, Moher D, Page MJ, Koffel JB, PRISMA-S Group (2021). PRISMA-S: an extension to the PRISMA statement for reporting literature searches in systematic reviews. Systematic Reviews.

[bib34] Serghiou S, Contopoulos-Ioannidis DG, Boyack KW, Riedel N, Wallach JD, Ioannidis JPA (2021). Assessment of transparency indicators across the BIOMEDICAL literature: how open is open. PLOS Biology.

[bib35] Sukhtankar S (2017). Online Appendix for Replications in Development Economics.

[bib36] Tricco AC, Lillie E, Zarin W, O’Brien KK, Colquhoun H, Levac D, Moher D, Peters MDJ, Horsley T, Weeks L, Hempel S, Akl EA, Chang C, McGowan J, Stewart L, Hartling L, Aldcroft A, Wilson MG, Garritty C, Lewin S, Godfrey CM, Macdonald MT, Langlois EV, Soares-Weiser K, Moriarty J, Clifford T, Tunçalp Ö, Straus SE (2018). PRISMA extension for Scoping reviews (PRISMA-SCR): checklist and explanation. Annals of Internal Medicine.

[bib37] Wallach JD, Boyack KW, Ioannidis JPA (2018). Reproducible research practices, transparency, and open access data in the biomedical literature, 2015–2017. PLOS Biology.

